# Occurrence and molecular characterization of cyst nematode species (*Globodera* spp.) associated with potato crops in Colombia

**DOI:** 10.1371/journal.pone.0241256

**Published:** 2021-07-14

**Authors:** Daniela Vallejo, Diego A. Rojas, John A. Martinez, Sergio Marchant, Claudia M. Holguin, Olga Y. Pérez

**Affiliations:** 1 Sede Medellín, Universidad Nacional de Colombia, Medellín, Antioquia, Colombia; 2 Centro de Investigación Tibaitatá, Corporación Colombiana de Investigación Agropecuaria AGROSAVIA, Mosquera, Cundinamarca, Colombia; 3 Escuela de biología, Universidad Industrial de Santander, Bucaramanga, Santander, Colombia; 4 Centro de Investigación La Selva, Corporación Colombiana de Investigación Agropecuaria AGROSAVIA, Rionegro, Antioquia, Colombia; University of Limpopo, SOUTH AFRICA

## Abstract

Potato cyst nematodes (PCN) from the genus *Globodera* spp. cause major losses in the potato (*Solanum tuberosum)* industry worldwide. Despite their importance, at present little is known about the status of this plant pathogen in cultivated potatoes in Colombia. In this study, a total of 589 samples collected from 75 geographic localities in nine potato producing regions of Colombia (Cundinamarca, Boyacá, Antioquia, Nariño, Santander, Norte de Santander, Tolima, Caldas and Cauca) were assayed for the presence of potato cyst nematodes. Fifty-seven percent of samples tested positive for PCN. Based on phylogenetic analysis of the internal transcribed spacer region (ITS1-5.8S-ITS2) of the rRNA gene and D2-D3 expansion segments of the 28S rRNA gene, all populations but one were identified as *Globodera pallida*. Sequences of *G*. *pallida* from Colombia formed a monophyletic group closely related to Peruvian populations, with the lowest average number of nucleotide substitutions per site (*Dxy* = 0.002) and net nucleotide substitutions per site (*Da =* 0.001), when compared to *G*. *pallida* populations from Europe, South and North America. A single sample formed a well-supported subclade along with *G*. *rostochiensis* and *G*. *tabacum* from Japan, USA and Argentina. To our knowledge this is the first comprehensive survey of *Globodera* populations from Colombia that includes genetic data. Our findings on species diversity and phylogenetic relationships of *Globodera* populations from Colombia may help elucidate the status and distribution of *Globodera* species, and lead to the development of accurate management strategies for the potato cyst nematodes.

## Introduction

The cyst nematodes, *Globodera* Skarbilovich, 1959, are one of the most limiting plant parasitic nematodes around the world [1]. Within the genus, thirteen species have been identified, of which *G*. *rostochiensis*, *G*. *pallida*, *G*. *ellingtonae*, and *G*. *tabacum* are important for agriculture [2]. The potato cyst nematodes (PCN), *Globodera rostochiensis* (golden or yellow potato cyst nematode) and *Globodera pallida* (pale potato cyst nematode) cause major losses in potato (*Solanum tuberosum* L.) crops [3], and are also considered as official control pests in many countries [4]. These species cause damage to the potato plants, by penetrating and feeding into the root tissue, which causes nutritional and water deficiency that is expressed in chlorosis and wilting of the leaves, and may also cause low growth, dwarfism and proliferation of small lateral roots that lead to yield reduction [4]. If PCN species are left uncontrolled they may reduce potato yield up to 80% [5,6], representing major economic losses in the potato industry worldwide.

Identification of *Globodera* species based on morphological characterization of the perineal area of cysts (e.g. distance from vulva and anus and Granek’s ratio) and some characters of the second stage juvenile (e.g. stylet length and stylet knob shape) [4,7] may be ambiguous. Morphometric measurements of these characters often show overlap among species, making morphological identification of cyst nematodes time consuming and difficult, especially when differentiating *G*. *pallida* from *G*. *rostochiensis* and *G*. *tabacum* species complex **(***G*. *tabacum tabacum*, *G*. *tabacum solanacearum* and *G*. *tabacum virginiae*) [8,9]. Therefore, molecular diagnosis is a necessary and recommended complement to identify cyst nematode species [4].

For plant-parasitic nematodes, molecular diagnostics not only improve speed and accuracy of nematode identification, but also have allowed a better understanding of the biology of nematodes as agricultural pests [10]. The genomic regions more often used to study phylogenetic relationships for plant-parasitic nematodes include DNA fragments from the 28S ribosomal DNA (rDNA), internal transcribed spacer (ITS), as well as mitochondrial DNA (mtDNA) [2,10–15]. Ribosomal genes exhibit enough conserved inter-specific neutral genetic variation as to inform species delimitation without being prone to marker saturation [15–18]. For cyst nematodes identification, although several methods have been used, DNA-based approaches have shown to be more accurate to separate *G*. *pallida* from *G*. *rostochiensis* and other *Globodera* species and, ribosomal regions have also shown to be useful markers to distinguish species within the genus [12,17,19–21]. For new occurrences of *Globodera* spp., sequencing of DNA fragments is also recommended, especially for regions where genetic data has not been reported before and for PCN species that may not follow a typical profile [17,22]. For *Globodera* species from Colombia, genetic information including validation of currently available diagnostic DNA markers and molecular phylogenetics have not been documented.

In Colombia, *G*. *pallida* was first identified based on morphological characters in 1970 in Cumbal (municipality located in the Nariño department), at the south west extreme of the country [23]. In 1971, the species was regulated under the authority of The Instituto Colombiano Agropecuario (ICA) and listed as quarantine pest, limiting the access to export potato seeds from Nariño and its neighbor department, Cauca, to other potato producing regions of Colombia. In 1983, Nieto [24] conducted an intensive PCN survey and reported *G*. *pallida* in other municipalities of Nariño (Túquerres, Pupiales, Ipiales, Gualmatán, Sapuyes, among others), as well as in Cauca (Totoró, Cajibío, Silvia, Popayán, Páez, among others), with an average of 50–80 cysts/100 g of soil in Nariño and 9–10 cysts/100 g of soil in Cauca. The authors also sampled the nematode in Cundinamarca and Boyacá, the main potato producing departments in Colombia, and other minor producing potato regions such as Caldas, Tolima, Valle del Cauca, Santander and Norte de Santander, but only reported the presence of PCN in Nariño and Cauca. In 2004, the species was no longer listed as an official control pest. Yet, in a survey conducted from 2011 to 2012, PCN was reported in 12 out of 14 sampled fields in Tunja, Samacá and Ventaquemada (municipalities of Boyacá department) and Tausa, Tabio and Zipaquirá (municipalities of Cundinamarca department), although population densities were not registered [25]. Therefore, PCN was considered as a re-emerging pathogen in 2012 by the Federación Colombiana de Productores de Papa (Colombian Federation of Potato Producers–FEDEPAPA), and ICA [25].

To obtain better knowledge about *Globodera* spp. associated with potato crops in Colombia, it is necessary to develop DNA sequence information to better characterize populations from different geographic regions and to understand their distribution patterns. This information will also serve as a foundation to the design of effective control measures that require fast and accurate identification of species, and it is a crucial factor when searching for possible sources of host-plant resistance as well as for other management strategies. Therefore, the objectives of this study were to: i) survey the *Globodera* spp. populations detected in cultivated potatoes in Colombia; ii) carry out a molecular characterization of these *Globodera* populations based on sequences of the ITS1 of rRNA, partial 18S rRNA and, D2-D3 expansion segments of the 28S nuclear ribosomal RNA gene; and iii) study the phylogenetic relationships of *Globodera* spp. from Colombia by comparison with previously published molecular data of populations from other regions of the world.

## Materials and methods

### Ethics statement

Nematode sampling was performed under a collection permit granted by the Autoridad Nacional de Licencias Ambientales (ANLA) [Colombian National Authority Environmental Permits]: “Permit for collecting specimens of wild species of the biological diversity for non-commercial scientific research purposes”, resolution No. 1466, expedited on December 3, 2014.

### Nematode populations and sampling

**Fig 1:**
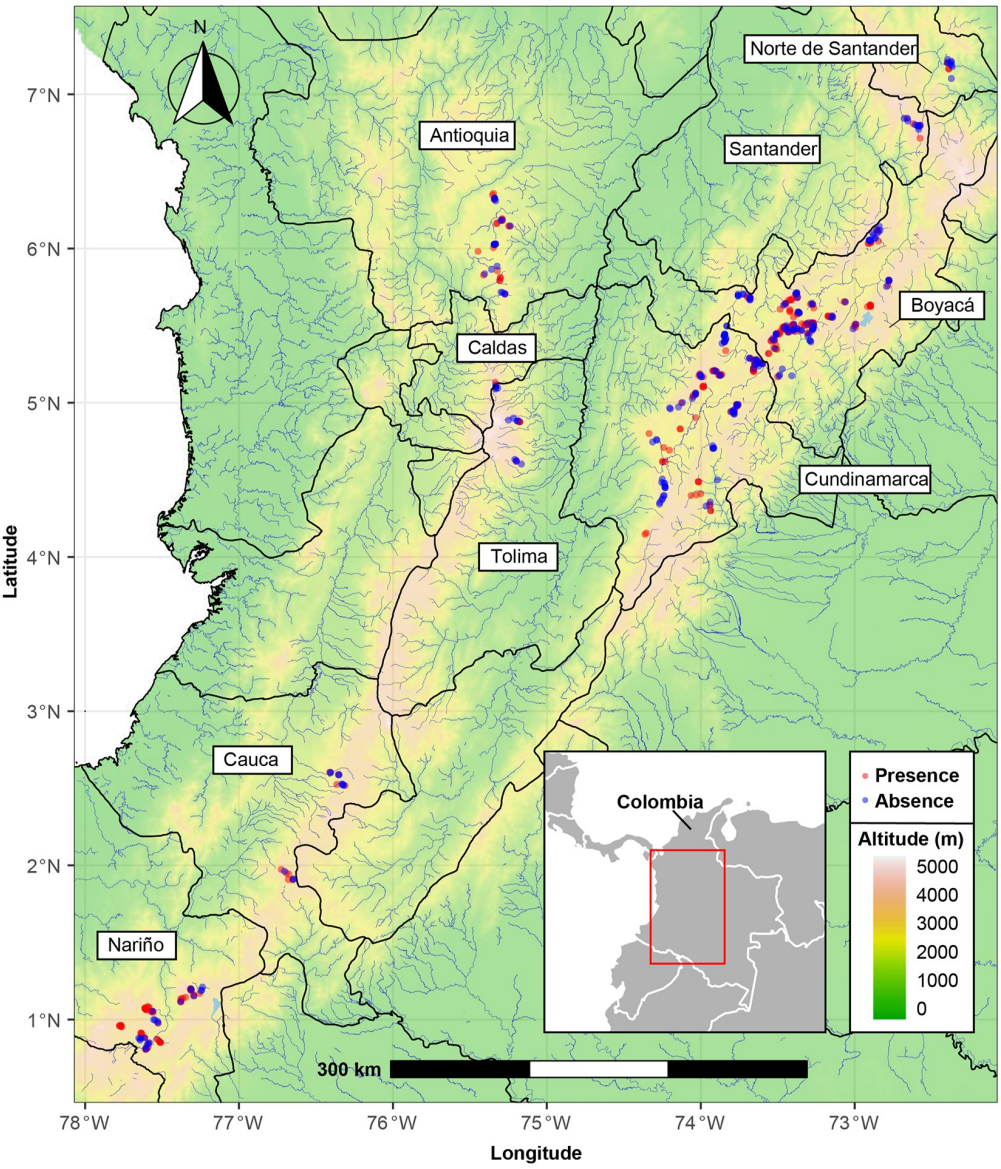
Map of the Colombian Andes showing sampling sites of PCN associated with potato crops in Colombia. Black lines represent department limits, blue lines represent rivers and lakes. Colors according to elevation map. Red dots mark the position of the sample sites that tested positive for PCN and blue dots mark the position of the sampling sites that tested negative for PCN. This figure has been adapted from a base map and data from OpenStreetMap and OpenStreetMap Foundation, original copyright 2020. ©OpenStreetMap contributors. Please notice that this figure is similar but not identical to the original image and is therefore for illustrative purposes only.

From 2013 to 2017, an extensive survey was conducted throughout the main commercial potato producing regions of Colombia. A total of 589 sampling sites were selected in 75 geographic localities using a stratified sampling strategy. The strata were defined as the departments (country subdivisions) with the highest potato growing area reported in Colombia [26], for a total of nine departments sampled: Cundinamarca, Boyacá, Antioquia, Nariño, Santander, Norte de Santander, Tolima, Caldas and Cauca (Fig 1, Table 1). At each department, the number of fields sampled per municipality was proportional to the potato area planted and fields at each municipality were selected based on established potato crops in pre-flowering and flowering stages (Fig 1, Table 1). Soil samples at each field were collected from within rows, at roughly equal intervals in a line transect pattern across an area of 10,000 m^2^ or less. A soil sample consisted of 60 soil cores (1.5 cm in diameter by 5 cm deep) taken near the root of the plants. Infected roots and surrounding soil of samples collected from each field were pooled into one composite sample. Samples were placed into plastic bags, transported to the laboratory of microbiology at the Corporación Colombiana de Investigación Agropecuaria (AGROSAVIA), Tibaitatá Research Center, in Mosquera, Cundinamarca, and stored at 4°C until processing. Cyst nematodes were extracted from soil samples using the Fenwick’s method [27], and cyst individuals per 100 cm^3^ of soil were counted and morphologically identified using the keys by Handoo et al. and Golden [7,28]. Population density was calculated as the average number of cysts per 100 cm^3^ of soil, population range as the minimum and maximum densities of cysts per 100 cm^3^ of soil; prevalence (%) was estimated by the formula: number of samples with cysts/ total number of samples collected)*100. Additionally, a viability test was performed by randomly selecting 10 cyst per population that were crushed using a Huijsman’s homogenizer [29] to release eggs and juveniles, alive eggs and j1 were counted under the stereoscope and viability percentage was calculated per population.

**Table 1:** *Globodera* species, density levels, and prevalence (%) in cultivated potato crops from the main growing regions in Colombia.

Department	Municipality	Species	# of samples collected	# of samples with *Globodera* spp.	Prevalence^a^ (%)	Average^b^/100 cm^3^ soil	Population range^c^/100 cm^3^ soil	Viability^d^ average (%)	Potato variety
Nariño	Túquerres	*G*. *pallida*	12	12	2.04	97.3	1–345	60	Diacol Capiro
Nariño	Ipiales	*G*. *pallida*	10	5	0.85	9.1	2–45	41	Diacol Capiro
Nariño	Pasto	*G*. *pallida*	15	9	1.53	14.7	1–64	60	Diacol Capiro
Nariño	Yacuanquer	*G*. *pallida*	2	2	0.34	3.5	3–4	45	Criolla
Nariño	Tangua	*G*. *pallida*	5	4	0.68	11	1–34	55	Diacol Capiro
Nariño	Ospina	*G*. *pallida*	5	4	0.68	47.4	5–93	38	Diacol Capiro
Nariño	Iles	*G*. *pallida*	5	1	0.17	16	1–80	59	Diacol Capiro
Nariño	Guachucal	*G*. *pallida*	7	7	1.19	21.3	1–84	63	Betina
Nariño	Pupiales	*G*. *pallida*	11	6	1.02	5.9	1–29	30	Diacol Capiro
Nariño	Córdoba	*G*. *pallida*	7	7	1.19	9.4	1–27	44	Pastusa Suprema
Cundinamarca	Chipaque	*G*. *pallida*	4	4	0.68	68.3	2–168	51	Pastusa Suprema
Cundinamarca	Madrid	*G*. *pallida*	5	2	0.34	1.8	2–7	63	Diacol Capiro
Cundinamarca	Pasca	-	6	0	-	-	-	-	Pastusa Suprema
Cundinamarca	Sesquilé	*G*. *pallida*	9	5	0.85	10.2	1–25	62	Pastusa Suprema
Cundinamarca	Subachoque	*G*. *pallida*	5	1	0.17	4	0–20	52	Pastusa Suprema
Cundinamarca	Tausa	*G*. *pallida*	25	17	2.89	84.2	1–1327	51	Pastusa Suprema
Cundinamarca	Ubaque	*G*. *pallida*	4	2	0.34	1.8	3–4	58	Superior
Cundinamarca	Ubaté	*G*. *pallida*	3	3	0.51	443.3	268–556	51	Parda Pastusa
Cundinamarca	Une	*G*. *pallida*	3	3	0.51	3.3	1–5	72	Criolla
Cundinamarca	Villapinzón	*G*. *pallida*	29	18	3.06	23.4	1–475	74	Pastusa Suprema
Cundinamarca	Zipaquirá	*G*. *pallida*	9	5	0.85	2	2–10	64	Pastusa Suprema
Cundinamarca	Cajicá	*G*. *pallida*	1	1	0.17	1	0–1	80	Diacol Capiro
Cundinamarca	Tenjo	*G*. *pallida*	2	2	0.34	72.5	50–95	76	Diacol Capiro
Cundinamarca	Sibaté	-	8	0	-	-	-	-	Criolla
Cundinamarca	Soacha	*G*. *pallida*	3	3	0.51	1	0–1	76	Diacol Capiro
Cundinamarca	Cogua	*G*. *pallida*	4	4	0.68	1.5	1–2	73	Betina
Cundinamarca	San Bernardo	*G*. *pallida*	2	2	0.34	2.5	2–3	51	Criolla
Cundinamarca	Fosca	*G*. *pallida*	4	2	0.34	2	0–5	69	Pastusa Suprema
Cundinamarca	Lenguazaque	*G*. *pallida*	3	1	0.17	1.7	0	60	Parda Pastusa
Cundinamarca	Simijaca	-	2	0	-	-	-	-	Pastusa Suprema
Cundinamarca	Susa	*G*. *pallida*	15	1	0.17	2.7	1–125	55	Pastusa Suprema
Cundinamarca	Guatavita	*G*. *pallida*	11	4	0.68	11.8	1–125	55	Pastusa Suprema
Cundinamarca	La Calera	-	5	0	-	-	-	-	Pastusa Suprema
Cundinamarca	Chocontá	*Globodera* sp.	8	7	1.19	43	1–93	70	Pastusa Suprema
Boyacá	Tota	*G*. *pallida*	6	4	0.68	4.7	2–19	58	Parda Pastusa
Boyacá	Toca	*G*. *pallida*	10	7	1.19	27.3	5–122	51	Tocarreña
Boyacá	Tunja	*G*. *pallida*	25	17	2.89	47.3	1–411	60	Diacol Capiro
Boyacá	Chíquiza	*G*. *pallida*	11	9	1.53	47	1–142	23	Betina
Boyacá	Úmbita	*G*. *pallida*	5	1	0.17	2	0–2	20	Tocarreña
Boyacá	Samacá	*G*. *pallida*	29	22	3.74	53.2	1–429	24	Diacol Capiro
Boyacá	Oicatá	*G*. *pallida*	6	4	0.68	7.7	1–36	24	Diacol Capiro
Boyacá	Sogamoso	*G*. *pallida*	8	8	1.36	107.5	1–305	38	Parda Pastusa
Boyacá	Siachoque	*G*. *pallida*	27	20	3.40	64	1–364	54	Parda Pastusa
Boyacá	Arcabuco	*G*. *pallida*	10	5	0.85	122.5	1–873	23	Parda Pastusa
Boyacá	Ventaquemada	*G*. *pallida*	15	12	2.04	7.9	1–73	53	Pastusa Suprema
Boyacá	Motavita	*G*. *pallida*	8	4	0.68	2.6	1–14	54	Betina
Boyacá	Sora	*G*. *pallida*	3	3	0.51	185	101–286	60	Diacol Capiro
Boyacá	Saboyá	*G*. *pallida*	16	5	0.85	1.4	1–15	36	Parda Pastusa
Boyacá	Soracá	*G*. *pallida*	9	8	1.36	84.7	1–306	24	Diacol Capiro
Boyacá	Boyacá	*G*. *pallida*	3	1	0.17	13.7	1–41	35	Diacol Capiro
Boyacá	Viracachá	*G*. *pallida*	3	2	0.34	1.3	1–2	32	Rubí
Boyacá	Ciénega	-	4	0	-	-	-	-	Pastusa Suprema
Boyacá	Mongua	*G*. *pallida*	2	1	0.17	0.5	0–1	38	ICA Única
Boyacá	Firavitoba	*G*. *pallida*	3	2	0.34	1	1–2	44	Parda Pastusa
Boyacá	Gámeza	*G*. *pallida*	5	2	0.34	0.8	1–2	20	Tocarreña
Boyacá	Belén	*G*. *pallida*	11	7	1.19	122.3	1–757	40	Parda Pastusa
Boyacá	Tutazá	*G*. *pallida*	12	3	0.51	6.9	1–78	49	ICA Única
Cauca	San Sebastián	*G*. *pallida*	8	5	0.85	4.1	1–13	48	Parda Pastusa
Cauca	Silvia	*G*. *pallida*	6	2	0.34	0.3	0–1	47	Criolla
Cauca	Totoró	*G*. *pallida*	6	2	0.34	4.3	1–23	51	Criolla
Antioquia	San Vicente	*G*. *pallida*	9	5	0.85	7.11	2–24	40	Criolla
Antioquia	La Unión	*G*. *pallida*	9	3	0.51	6	1–51	32	Diacol Capiro
Antioquia	Sonsón	*G*. *pallida*	9	5	0.85	7.5	1–29	16	Criolla
Antioquia	La Ceja	*G*. *pallida*	1	1	0.17	7	7–7	20	Criolla
Antioquia	Abejorral	*G*. *pallida*	6	3	0.51	2.17	1–7	15	Criolla
Antioquia	Marinilla	*G*. *pallida*	7	5	0.85	5.71	1–26	20	Criolla
Antioquia	Santuario	*G*. *pallida*	3	2	0.34	1.33	1–3	20	ICA Nevada
Norte de Santander	Pamplona	*G*. *pallida*	8	2	0.34	0.9	1–4	37	Criolla
Norte de Santander	Cácota	*G*. *pallida*	4	2	0.34	1	1–2	16	Criolla
Santander	Concepción	*G*. *pallida*	8	2	0.34	0.9	1–6	20	Parda Pastusa
Santander	Carcasí	*G*. *pallida*	2	1	0.17	0.5	0–1	36	Parda Pastusa
Santander	Cerrito	*G*. *pallida*	4	1	0.17	1	1–4	31	ICA Única
Caldas	Manizales	*G*. *pallida*	7	1	0.17	0.43	1–3	15	Parda Pastusa
Tolima	Anzoátegui	*G*. *pallida*	5	1	0.17	0.4	1–2	29	Pastusa Suprema
Tolima	Murillo	*G*. *pallida*	7	1	0.17	0.2	0–1	19	Parda Pastusa

^a^ Prevalence: (Number of samples with cysts/ total number of samples collected)*100.

^b^ Population density: Average number of cysts per 100 cm^3^ of soil.

^c^ Population range: Minimum and maximum densities of cysts per 100 cm^3^ of soil.

^d^ Viability: Average of cysts with viable eggs per population.

### DNA extraction, polymerase chain reaction and sequencing

For molecular characterization, from the departments that showed the highest cyst nematode densities, soil samples taken per municipality were pooled according to proximity distance (1 km and 5 km range), resulting in one to two samples per municipality, for a total of 26 populations analyzed (Table 1). From each pooled population, DNA was extracted from individual cysts or juveniles using the "Sigma Extract-N-Amp Kit (XNAT2)" kit (Sigma, St. Louis, MO) according to the protocol reported by Ma et al. [30] at AGROSAVIA, La Selva Research Center, Rionegro, Antioquia. The number of individuals analyzed per population depended upon the cyst nematode density present in each soil sample. DNA was then stored at -20°C until used.

PCR amplification of two genomic regions were performed using 12.5 μl of the Extract-N-AmpTM Tissue PCR kit (Sigma), 1 μl of each primer, 4 μl of DNA and water to complete a volume of 25 μl. The rDNA primers used for PCR and DNA sequencing are listed in Table 2. The ITS region of ribosomal DNA was amplified using 94° C for 2.5 min for initial denaturation, followed by 40 cycles at 94° C for 1 min, 55° C for 1 min, 72° C for 2 min, and a final extension of 72° C for 5 minutes. For the 28S region, initial denaturation was 94° C for 5 min, 40 cycles of 94° C for 30 sec, 58° C for 30 sec, 72° C for 1 min, and a final extension of 72° C for 10 min [31]. The products were loaded on a 1.5% agarose gel and visualized using gel red (Biotium, San Francisco, CA). Sanger sequencing of the amplicons was performed in both directions by CorpoGen (Bogotá, Colombia).

**Table 2:** Primers used for polymerase chain reaction and DNA sequencing of *Globodera* spp. individuals recovered from cultivated potatoes in Colombia.

Primer	Marker	Sequence (5’ to 3’)	Reference
F194	ITS	CGTAACAAGGTAGCTGTAG	[32]
F195	ITS	TCCTCCGCTAAATGATATG	[33]
D2A	28S	ACAAGTACCGTGAGGGAAAGTTG	[33]
D3B	28S	TCGGAAGGAACCAGCTACTA	[34]

28S = Large ribosomal RNA subunit and ITS = internal transcribed spacer 1 and 2 including 5.8S rRNA.

### Sequence alignment and phylogenetic analyses

Resulting sequences were assembled in Sequencher^®^ software version 5.1 (Gene Codes Corporation, Ann Arbor, MI USA) and manually reviewed for base calling errors. Partial 28S rRNA and ITS1-2 + 5.8S rRNA gene sequences from *G*. *pallida*, *G*. *mexicana*, *G*. *rostochiensis*, *G*. *tabacum*, *G*. *ellingtonae*, and *G*. *artemisiae*, were retrieved from GenBank nucleotide database and included in the alignment (Table 3) [8,19,21,35–43]. Sequences of *Punctodera punctata* and *P*. *chalcoensis* also obtained from GenBank (AF274416.1, DQ328699.1.1, AY090885.1), were used as outgroup taxa for both gene regions. After that, sequence alignments were performed using Clustal W [44] and manually edited using BioEdit v 7.2 [45]. To remove ambiguous regions in the alignment the program Gblocks v.0.91b was used with the standard settings [46]. Newly generated sequences for both gene regions were deposited in GenBank (Table 3).

**Table 3:** Details of cyst nematode populations included in the molecular and phylogenetic studies from cultivated potatoes in Colombia and reported in other studies.

*Globodera* species	Location (Municipality, Department)	Specimen code	Accession number 28S	Accession number ITS
*Globodera pallida*	La Unión, AN*	A1A	MH389939	MH389979
*Globodera pallida*	La Unión, AN	A1B	MH389938	MH389978
*Globodera pallida*	La Unión, AN	A1C	-	MH389977
*Globodera pallida*	Ventaquemada, BO	B2A	MH389946	MH389986
*Globodera pallida*	Ventaquemada, BO	B2B	MH389945	MH389985
*Globodera pallida*	Ventaquemada, BO	B2C	MH389944	MH389984
*Globodera pallida*	Ventaquemada, BO	B2D	MH389943	MH389983
*Globodera pallida*	Arcabuco, BO	B3D	MH389942	MH389982
*Globodera pallida*	Chíquiza, BO	B4D	MH389937	-
*Globodera pallida*	Tunja, BO	B5D	-	MH389976
*Globodera pallida*	Toca, BO	B6D	MH389936	MH389975
*Globodera pallida*	Sogamoso, BO	B7A	MH389935	MH389974
*Globodera pallida*	Sogamoso, BO	B7B	MH389934	-
*Globodera pallida*	Sogamoso, BO	B7D	MH389933	-
*Globodera pallida*	Sora, BO	B8A	MH389932	MH389973
*Globodera pallida*	Sora, BO	B8C	-	MH389972
*Globodera pallida*	Sora, BO	B8D	-	MH389971
*Globodera pallida*	Samacá, BO	B9D	MH389941	MH389981
*Globodera pallida*	Soracá, BO	B11A	MH389931	-
*Globodera pallida*	Soracá, BO	B11B	MH389930	-
*Globodera pallida*	Soracá, BO	B11C	MH389929	-
*Globodera pallida*	Soracá, BO	B12A	MH389940	MH389980
*Globodera pallida*	Susa, CU	C1A	MH389928	MH389970
*Globodera pallida*	Susa, CU	C1B	MH389927	-
*Globodera pallida*	Susa, CU	C1D	-	MH389969
*Globodera pallida*	Guatavita, CU	C2B	MH389926	MH389968
*Globodera pallida*	Guatavita, CU	C2C	MH389925	MH389967
*Globodera pallida*	Guatavita, CU	C3C	MH389923	MH389965
*Globodera pallida*	Guatavita, CU	C3D	MH389922	-
*Globodera pallida*	Ubaté, CU	C4C	MH389921	-
*Globodera pallida*	Ubaté, CU	C4D	MH389920	-
*Globodera pallida*	Tausa, CU	C5A	MH389919	MH389964
*Globodera pallida*	Tausa, CU	C5B	MH389918	-
*Globodera pallida*	Subachoque, CU	C6B	MH389917	-
*Globodera pallida*	Sesquilé, CU	C8A	MH389916	-
*Globodera pallida*	Sesquilé, CU	C8C	MH389915	MH389963
*Globodera pallida*	Sesquilé, CU	C8D	-	MH389962
*Globodera pallida*	Cajicá, CU	C10D	-	MH389961
*Globodera pallida*	Villapinzón, CU	C11A	MH389914	MH389960
*Globodera pallida*	Villapinzón, CU	C11D	MH389913	MH389959
*Globodera pallida*	Túquerres, NA	N2A	MH389949	MH389990
*Globodera pallida*	Túquerres, NA	N2B	MH389948	MH389989
*Globodera pallida*	Túquerres, NA	N2C	-	MH389988
*Globodera pallida*	Túquerres, NA	N2D	MH389947	MH389987
*Globodera pallida*	Guachucal, NA	N4A	MH389912	-
*Globodera pallida*	Guachucal, NA	N4B	MH389911	-
*Globodera pallida*	Guachucal, NA	N4C	MH389910	MH389958
*Globodera pallida*	Guachucal, NA	N4D	MH389909	-
*Globodera pallida*	Belén, NA	N5A	-	MH389957
*Globodera pallida*	Belén, NA	N5B	-	MH389956
*Globodera pallida*	Belén, NA	N5C	MH389908	MH389955
*Globodera pallida*	Belén, NA	N5D	MH389907	MH389954
*Globodera pallida*	Ospina, NA	N8B	-	MH389953
*Globodera pallida*	Ospina, NA	N8C	MH389906	MH389952
*Globodera pallida*	Ospina, NA	N8D	MH389905	MH389951
*Globodera pallida*	Ipiales, NA	N9A	MH389904	MH389950
*Globodera sp*	Chocontá, CU	C3A	MH389924	MH389966
*Globodera pallida*	Peru	-	-	GU084813.1
*Globodera pallida*	Peru	-	-	GU084805.1
*Globodera pallida*	Chile	-	-	GU084800.1
*Globodera pallida*	Ukraine	-	-	AJ606687.1
*Globodera pallida*	England	-	-	DQ847110.1
*Globodera pallida*	Poland	-	-	EU855119.1
*Globodera pallida*	Peru	-	-	GU084804.1
*Globodera pallida*	Peru	-	-	GU084806.1
*Globodera pallida*	Peru	-	-	HQ670269.1
*Globodera mexicana*	Mexico	-	-	EU006707.1
*Globodera mexicana*	Mexico	-	-	EU006708.1
*Globodera rostochiensis*	USA	-	-	EF153839.1
*Globodera rostochiensis*	Australia	-	-	EF622524.1
*Globodera rostochiensis*	Canada	-	-	FJ212166.1
*Globodera rostochiensis*	England	-	-	EF153840.1
*Globodera rostochiensis*	Bolivia	-	-	GU084809.1
*Globodera tabacum*	Japan	-	-	AB207272.1
*Globodera tabacum*	USA	-	-	GQ294525.1
*Globodera tabacum*	USA	-	-	DQ847112.1
*Globodera tabacum*	Argentina	-	-	DQ097515.2
*Globodera ellingtonae*	USA	-	-	GQ896543.1
*Globodera ellingtonae*	Chile	-	-	GU084808.1
*Globodera ellingtonae*	USA	-	-	DQ097514.2
*Punctodera punctata*	Belgium	-	-	AF274416.1
*Punctodera chalcoensis*	Mexico	-	-	AY090885.1
*Globodera pallida*	France	-	KJ409636.1	-
*Globodera pallida*	Slovakia	-	KJ409626.1	-
*Globodera pallida*	England	-	JN712219.1	-
*Globodera pallida*	Chile	-	JN712220.1	-
*Globodera pallida*	France	-	GU338021.1	-
*Globodera ellingtonae*	USA	-	JN712217.1	-
*Globodera tabacum*	USA	-	GQ294492.1	-
*Globodera rostochiensis*	Canada	-	JN712223.1	-
*Globodera rostochiensis*	Slovakia	-	KJ409625.1	-
*Globodera artemisiae*	Hungary	-	KU845472.1	-

28S = Large ribosomal RNA subunit and ITS = internal transcribed spacer 1 and 2 including 5.8S rRNA.

Phylogenetic relationships among partial sequences of the 28S rRNA and Internal Transcribed Spacer 1 and 2 plus 5.8S rRNA genes were inferred using Bayesian Inference (BI) and Maximum Likelihood (ML) methods. For both gene regions, PartitionFinder v.2.0 [47] was used to select the best-fit partitioning scheme and models of evolution for phylogenetic analyses according to the Greedy algorithm using the Bayesian Information Criterion (BIC). The sequence partition for 28S rRNA gene was a single partition with HKY substitution model [48] for each of the 3 positions of the codon. The best sequence partition for ITS1-2 + 5.8S rRNA gene internal transcribed spacer 1 and 2 including the 5.8S rRNA region was a partition that included the first and second position of the codon with a K80 model [49] substitution model, and a second partition that consisted on the third codon position under the K80 with proportions of invariable sites (K80+I). Bayesian Inference analyses were performed using MrBayes v.3.1.2 [50], with five independent runs of four Markov chains for 1 x 10^6^ generations and default heating values, sampling every 100 generations with 2500 samples discarded as burn-in after checking for convergence. Clades were considered strongly supported when values were > 0.95 [51]. For Maximum likelihood analyses the software GARLI v.2.0 [52] was used. Bootstrap support for trees was generated with 1,000 replicate searches and summarized in a consensus tree using SumTrees [53], clades were considered as well/strongly supported when bootstrap was >70%. In addition, in order to characterize the genetic divergence between cyst nematodes from Colombia and *Globodera* species already reported, the average number of nucleotide substitution per site (*Dxy*), net nucleotide substitutions per site (*Da*) and number of fixed differences (*Fd*) among genetic groups were computed using DnaSP v.6.12.03 [54].

## Results

### Field survey

**Fig 2:**
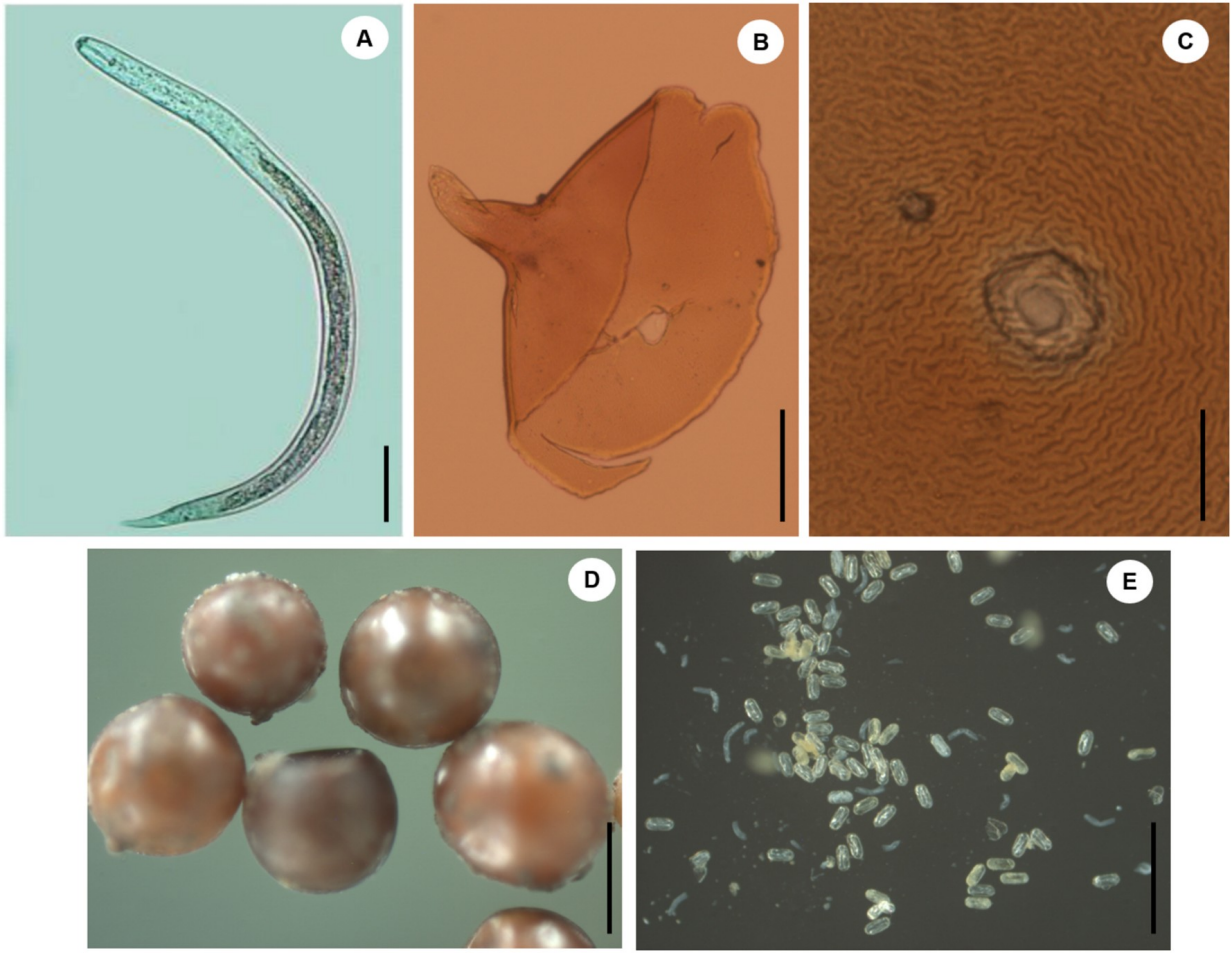
Light micrographs of *Globodera pallida* from Colombia (Population B2: Ventaquemada, Boyacá). A) Second-stage juvenile. B) Anterior region. C) Cysts. D) Eggs. Scale bars: A-C = 50 μm; D, E = 100 μm.

Of the 589 potato fields sampled, cyst nematodes were detected in 355 fields distributed in 69 municipalities of Colombia, with densities ranging from 1 to 1,327 cysts per 100 cm^3^ of soil (Table 1). The predominant species was *G*. *pallida* (Fig 2), identified in 51% of the fields sampled in Cundinamarca, 63.3% in Boyacá, 72.2% in Nariño, 54% in Antioquia, 45% in Cauca, 33% in Norte de Santander, 29% in Santander, 17% in Tolima and 14% in Caldas, with densities ranging from 1 to 1327 cysts/100 cm^3^ of soil, 1 to 873 cysts/100 cm^3^ of soil, 1 to 345 cysts/100 cm^3^ of soil, 1 to 51 cysts/100 cm^3^ of soil, 1 to 23 cysts/100 cm^3^ of soil, 1 to 4 cysts/100 cm^3^ of soil, 1 to 6 cysts/100 cm^3^ of soil, 1 to 3 cysts/100 cm^3^ of soil and 1 to 2 cysts/100 cm^3^ of soil, respectively. Among municipalities, the highest mean densities were detected in Ubaté (443.3 cysts/100 cm^3^ of soil), followed by Sora, Arcabuco, Belén, Sogamoso, Túquerres and Tausa (185, 122.5, 122.3, 107.5, 97.3 and 84,2 cysts/100 cm^3^ of soil, respectively). Cyst nematodes were not found in Pasca, Sibaté, Simijaca, and La Calera in Cundinamarca, nor in Ciénega in Boyacá (Table 1). All samples positive for PCN showed cysts with viable eggs, and viability percentage ranged from 15 to 80%. Boyacá and Cundinamarca were the departments that had in average the higher viability (Table 1).

Given host-based grouping, PCN was detected in samples taken from varieties of *Solanum tuberosum* Group *Andigena* such as Diacol Capiro (107 out of 158 samples), Betina (24 out of 30 samples), Pastusa Suprema (81 out of 167 samples), Parda Pastusa (66 out of 105 samples), Tocarreña (10 out of 20 samples), Rubí (2 out of 3 samples), ICA Única (4 out of 16 samples), ICA Nevada (2 out of 3 samples), Superior (2 out of 10) and, from varieties of *Solanum tuberosum* Group *Phureja* such as Criolla variety (32 out of 69 samples) (Table 1). A morphologically different species (under description), was only detected in one field in Chocontá (Cundinamarca) on Suprema variety (Table 1).

### Molecular analysis

The amplification of D2-D3 expansion segments of 28S rRNA and internal transcribed spacer 1, 2 including the 5.8S rRNA yielded single fragments of 609 and 848 bp, respectively. Forty-two new D2-D3 of 28S rRNA gene sequences and twenty-eight new internal transcribed spacer 1 and 2 including the 5.8S rRNA were obtained in the present study (Table 2).

**Fig 3:**
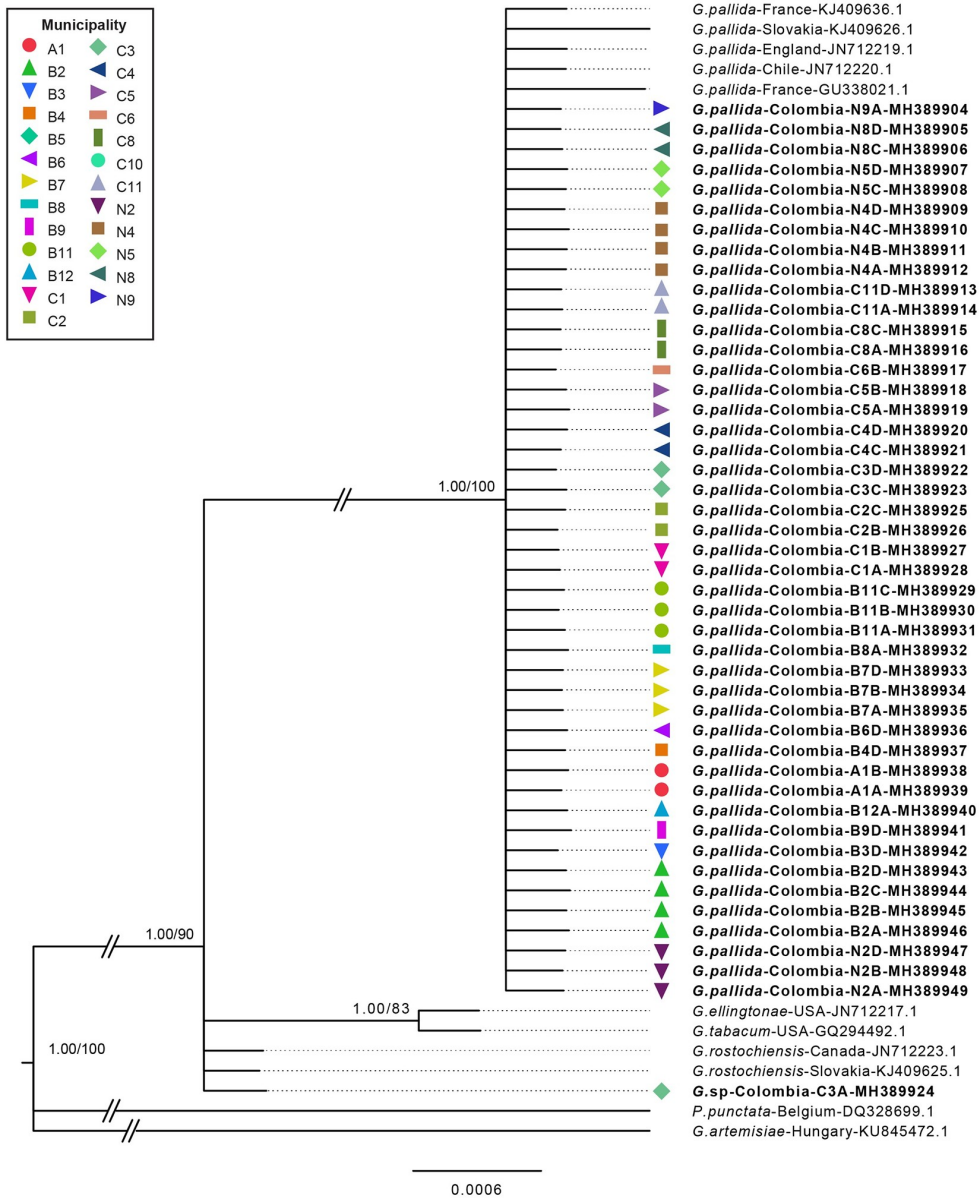
Phylogenetic relationships within the genus *Globodera*. Bayesian 50% majority rule consensus trees as inferred from D2–D3 expansion segments of 28S rRNA as a single partition with HKY model. Node-support values: Left value posterior probability BI shown if >95%, right value bootstrap from ML analysis shown only if >70%. Newly obtained sequences in this study are in bold.

Phylogenetic relationships inferred from analyses of D2-D3 expansion segments of 28S rRNA of a multiple-edited alignment (57 sequences), showed two well supported major clades based on BI and ML inferences (PP = 1.00, BP = 90) (Fig 3). A highly supported clade (i) (PP = 1.00, BP = 90), was formed by sequences of *G*. *pallida* from France, Chile, England, Slovakia and all but one cyst nematode sequence obtained in this study from Colombia. The second major clade, Clade (ii) grouped three species, *G*. *ellingtonae* and *G*. *tabacum* that formed a well-supported subclade (PP = 1.00, BP = 83) clearly separated from a politomy formed by a single cyst nematode sample (*G*. sp) from Colombia and *G*. *rostochiensis* from Canada and Slovakia (Fig 3).

**Fig 4:**
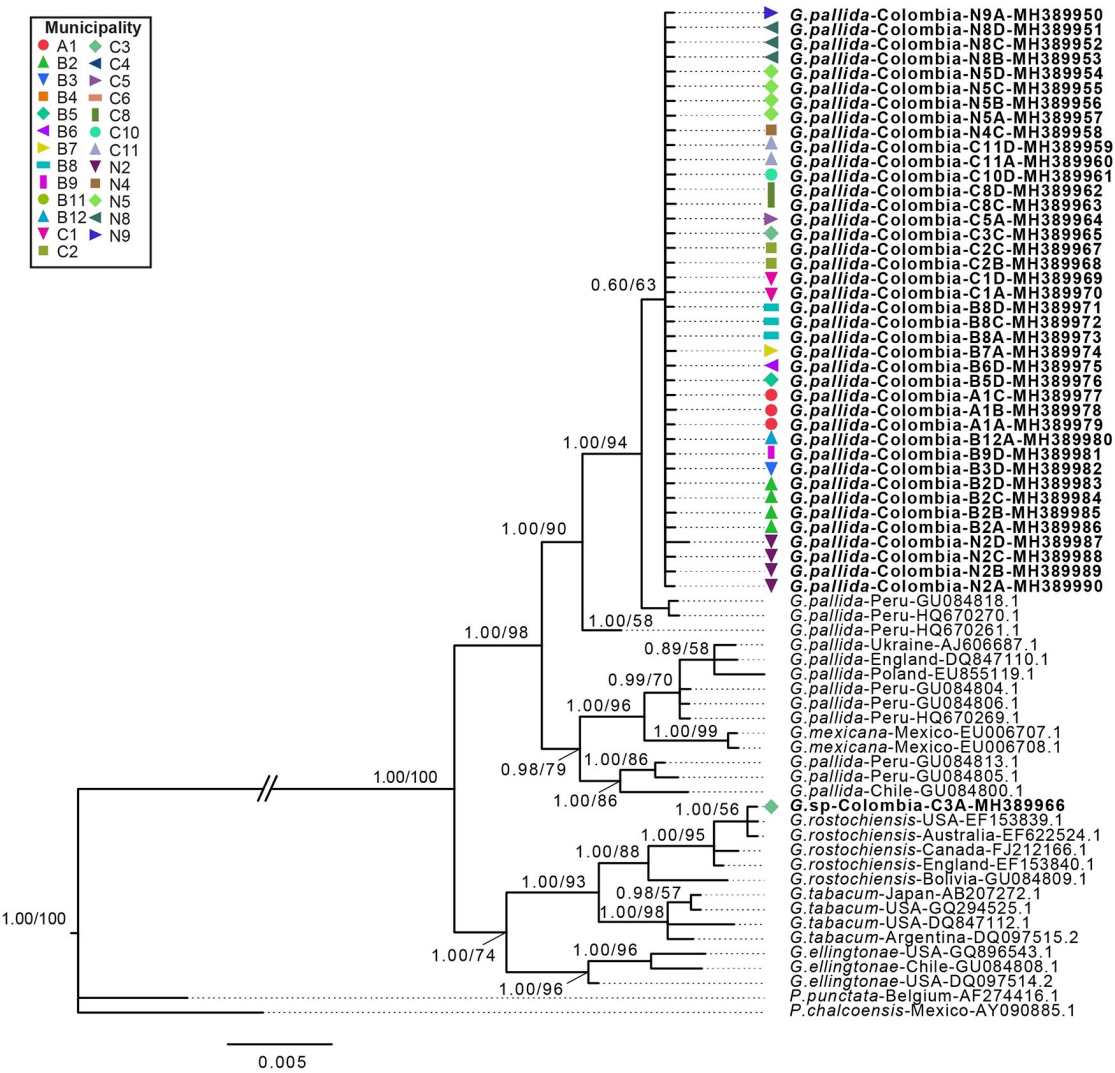
Phylogenetic relationships within the genus *Globodera*. Bayesian 50% majority rule consensus trees as inferred from Internal Transcribed Spacer 1 and 2 plus 5.8S rRNA gene with first and second position with K80 substitution model, and a second partition with the third codon under K80+I model. Node-support values: Left value posterior probability BI shown if >95%, right value bootstrap from ML analysis shown only if >50%. Newly obtained sequences in this study are in bold.

The 50% majority-rule BI consensus tree of the alignment generated for the 69 sequences of the region conformed by the internal transcribed spacer 1 and 2 including the 5.8S rRNA regions, showed two well supported major clades (PP = 1.00/ BP = 100) that were consistent with the findings based on 28S rRNA phylogeny (Fig 4). Clade (i) was formed by *G*. *pallida* and *G*. *mexicana*, and Clade (ii) was formed by *G*. *rostochiensis*, *G*. *tabacum*, *G*. *ellingtonae* and one sequence from Colombia. In Clade (i) two sequences of *G*. *pallida* from Peru along with the rest of sequences from Colombia formed a well-supported subclade (PP = 1.00, BP = 90) that was clearly separated (PP = 1.00, BP = 94) from one sequence of *G*. *pallida* from Peru. The sister clade of this sub-clade was formed by other sequences of *G*. *pallida* from Peru and European countries such as Ukraine, England and Poland, that were separated with high support (PP = 1.00, BP = 96) from sequences of *G*. *mexicana* and moderately support (PP = 1.00, BP = 79) of *G*. *pallida* from Chile and Peru. In Clade (ii) a single sequence from Colombia formed a well-supported subclade (PP = 1.00, BP = 93) along with *G*. *rostochiensis* from USA, Australia, Canada, England and Bolivia that was related with *G*. *tabacum* from Japan, USA and Argentina. This subclade formed a sister clade with *G*. *ellingtonae* from USA and Chile with high support (PP = 1.00, BP = 74).

Genetic distances among cyst nematodes sequences from Colombia and other nematodes species included in the phylogenetic analyses are summarized in Table 4. Based on the 28S rRNA gene sequences, all sequences from Colombia from Clade (i) had the lowest average number of nucleotide substitutions per site (Nucleotide divergence—*Dxy* = 0.002) and lowest number of net nucleotide substitutions per site (net genetic distance—*Da* = 0.001) when compared to *G*. *pallida* sequences from the other countries without fixed differences among groups (Table 4). The cyst nematode sequence from Colombia in Clade (ii) had the lowest divergence when compared with *G*. *rostochiensis* (*Dxy* = 0.001, *Da* = 0.000) without showing any fixed differences among groups (Table 4). In agreement with the 28S rRNA marker, the genetic distances based on the internal transcribed spacer 1 and 2 including the 5.8S rRNA gene sequences of cyst nematodes from Colombia in Clade (i) was lowest when compared with *G*. *pallida* (*Dxy* = 0.014, *Da* = 0.008), with one fixed substitution (Fig 3, Table 4) and lowest in Clade (ii) when compared with *G*. *rostochiensis* (*Dxy* = 0.003, *Da* = 0.000) with no fixed differences among groups (Table 4).

**Table 4:** Gene divergence between potato cyst nematodes from Colombia and other species retrieved from GenBank.

Marker	Group 1	Group 2	Dxy	Da	Fd
28S	*G*. *pallida* - Colombia	*G*. *pallida*	0.00227	0.00116	0
		*G*. *rostochiensis*	0.01072	0.00988	4
		*G*. *ellingtonae*	0.01370	0.01243	6
		*G*. *tabacum*	0.01443	0.01400	6
		*G*. *artemisiae*	0.02661	0.02618	15
28S	*G*. sp - Colombia	*G*. *pallida*	0.01165	0.01013	6
		*G*. *rostochiensis*	0.00125	0.00000	0
		*G*. *ellingtonae*	0.00498	0.00332	2
		*G*. *tabacum*	0.00415	0.00332	2
		*G*. *artemisiae*	0.02076	0.01993	12
ITS	*G*.*pallida* - Colombia	*G*. *pallida*	0.01418	0.00818	1
		*G*. *rostochiensis*	0.03188	0.02871	21
		*G*. *ellingtonae*	0.02973	0.02634	18
		*G*. *tabacum*	0.02721	0.02550	20
		*G*. *mexicana*	0.02035	0.02026	16
ITS	*G*.sp - Colombia	*G*. *pallida*	0.03230	0.02600	20
		*G*. *rostochiensis*	0.00395	0.00027	0
		*G*. *ellingtonae*	0.02582	0.02189	16
		*G*. *tabacum*	0.01675	0.01478	12
		*G*. *mexicana*	0.03289	0.03230	27

28S = Large ribosomal RNA subunit and ITS = internal transcribed spacer 1 and 2 including 5.8S rRNA.

Dxy, Da and Fd correspond to average number of nucleotide substitutions per site, the number of net nucleotide substitutions per site, and number of fixed differences between compared groups, respectively.

## Discussion

Even when PCN is considered a re-emerging potato pathogen in Colombia, first identified in the department of Nariño in 1970 [23], documented surveys only report PCN in municipalities of Nariño, Cauca, Boyacá and Cundinamarca [24,25]. Our comprehensive study that sampled 75 municipalities in 9 potato producing departments of Colombia found that 60% of the tested samples were positive for PCN (355 out of 589 sampled fields), and that the pathogen is widespread in all Colombian producing potato departments (Cundinamarca, Boyacá, Nariño, Antioquia, Cauca, Norte de Santander, Santander, Tolima and Caldas) (Fig 1), with cysts that contain viable eggs present in all sampled regions.

However, there was variation in population densities among regions. The highest densities were found in Cundinamarca and Boyacá, ranging from 1 to 1,327 cyts/100 g of soil and 1 to 873 cysts/100 g of soil, respectively. Nieto et al. (1983) [24] surveyed these departments in early 1980s, but since detected cysts were empty or contained non-viable eggs, these regions were declared as PCN free. Later, Arciniegas et al (2012) [25], reported PCN in Tunja, Samacá and Ventaquemada in Boyacá and Tausa, Tabio and Zipaquirá in Cundinamarca. In our study, PCN was detected in new municipalities with the highest densities found in Tausa, Ubaté and Villapinzón in Cundinamarca and, in Arcabuco, Belén, Tunja, Samacá, Sogamoso and Sora in Boyacá (Table 1). Boyacá and Cundinamarca are the largest producers of potatoes in Colombia, with 40,724 and 61,322 harvested hectares corresponding to 26% and 39% of the total potato national production in 2018, respectively [26]. Potatoes are the main plant crop grown by farmers and fields are usually planted in monocultures for several continuous cycles. As PCNs are highly specialized, sedentary and obligate endoparasites of solanaceous plants [1,22,55], the constant presence of potato crops in monocultures for several cycles may lead to persistence and increase of this plant pathogen in these two regions overtime. In contrast, in Nariño department, although the nematode was found in all municipalities sampled, PCN densities were lower, from 1 to 345 cysts/100 g of soil, and similar ranges were found by Nieto et al (1983) [24]. Nariño ranks third in production with 31,611 harvested hectares (19,35% of total potato national production), in contrast to Boyacá and Cundinamarca, in this department farmers usually grow different potato cultivars within a field (e.g. Diacol Capiro, Pastusa Suprema, Betina) with a crop rotation scheme usually with non-host plants such as corn, cabbage, lettuce, onion and pastures, and the use of biological microorganisms for the control of other pest problems has also implemented [56], which reduces pesticides use. Similar potato production scheme was observed in its neighbor department, Cauca, and PCN densities decreased in the latter from 23 cysts/100 g of soil [24] to 5,97 cysts/100 of soil in this study. Considering that *G*. *pallida* requires a living potato plant to complete its life cycle [6], the management practices implemented in both departments may reflect the reduction of PCN densities in these regions.

Our results also show *G*. *pallida* populations have spread into new regions of Colombia. In the departments of Antioquia, Caldas, Tolima, Santander and Norte de Santander, PCN was detected in all municipalities sampled, although with low population levels (5.97 cysts/100 of soil in average in Antioquia, 0.95 cysts/100 of soil in Norte de Santander, 0.8 cysts/100 of soil in Santander, 0.43 cysts/100 of soil in Caldas and 0.3 cysts/100 of soil in Tolima). To our knowledge, this is the first report of the presence of *G*. *pallida* in these departments. These regions represent in general, low potato growing areas with low participation in national potato production (2.27% in average in 2018) [26], and were therefore considered before as PCN free. The spread of PCN is mainly caused through tubers, soil or equipment contaminated with cysts [55] and potato seed tubers in these departments frequently come from Boyacá and Cundinamarca, which may allow the dissemination of this plant pathogen into these new regions. Nevertheless, population levels in these departments are low and the extent of PCN is limited, therefore, to maintain low levels and to avoid the spread into new areas, intensive monitoring program for PCN should be implemented in all potato producing regions of Colombia.

### Molecular identification and phylogenetic relationships of PCN species from Colombia

The molecular phylogeny of PCN populations based on ITS1-5.8S-ITS2 rDNA and 28S D2-D3 regions supported the presence of at least two PCN species in Colombia. *Globodera pallida* was found in all populations that resulted positive for PCN and, molecular phylogeny based on the ITS1-5.8S-ITS2 rDNA grouped all *G*. *pallida* from Colombia in a single clade that was closely related to P5A pathotype strains (GenBank accession number HQ670270.1) and clone La Libertad (GenBank accession number GU084818) (Fig 4). This finding suggests that *G*. *pallida* present in Colombia have a different origin than *G*. *pallida* present in countries such as Ukraine, England and Poland that cluster as a monophyletic clade with other Peruvian strains, and *G*. *pallida* present in Chile that cluster with a different Peruvian strain (Figs 3 and 4). Despite that P5A Peruvian strain has been considered as a different species [12,17], a recent study based on ITS rRNA, *COI* and *cytb* mitochondrial regions concluded that all clades within *G*. *pallida* belong to a single species [2]. The 28S D2-D3 phylogeny, although with lower level of resolution, also clustered all but one PCN populations from Colombia with *G*. *pallida* around the globe as a monophyletic clade (Fig 3). For both gene regions, a single sequence from the C3 population (Chocontá) grouped in a distant clade along with individuals of *G*. *rostochiensis*. Genetic distance analyses based on gene regions ITS1-5.8S-ITS2 and D2-D3, were congruent with these findings showing *G*. *pallida* from Colombia with the smallest *Da* (0.8% and 0.12% for ITS rDNA and D2-D3, respectively) and the smallest *Dxy* (1.41% and 0.23% for ITS rDNA and D2-D3, respectively) when compared with other *G*. *pallida* populations. Similarly, genetic distances from C3 population showed the lowest distance when compared with *G*. *rostochiensis* (*Dxy* = 0.001, *Da* = 0.000).

Therefore, the ITS1-5.8S-ITS2 rDNA and 28S D2-D3 molecular analyses were able to identify with high phylogenetic support *G*. *pallida* and *G*. *rostochiensis*. Additionally, ITS1-5.8S-ITS2 rDNA phylogenetic resolution supports a northern Peru origin of *G*. *pallida* present in Colombia, nevertheless this hypothesis must be further investigated using additional samples and molecular markers, with statistical inference such as model testing and coalescent demographic reconstruction. Although with less taxa included, molecular phylogeny based on 28S D2-D3 gene improved the node support found in previous phylogenies between *G*. *pallida* and *G*.*tabacum* (i.e. PP = 54 and 72%) (e.g., [9,16]), and the unresolved positions for *G*. *rostochiensis* [16]. Taken all together, both DNA markers used in this study showed to be useful to identify *Globodera* species present in Colombia, with ITS1-5.8S-ITS2 rDNA being more informative in phylogeographic perspective [12].

## Conclusions

This study provides new information about the status and prevalence of PCN species associated with cultivated potatoes in the main producing regions of Colombia including for the first time genetic information. Molecular phylogenies with ITS1-5.8S-ITS2 rDNA and D2/D3 28S regions were effective in the identification of *G*. *pallida*, the dominant species present in all departments surveyed in this study, and suggest the presence of *G*. *rostochiensis*, in one municipality of Cundinamarca, which is currently under description. Considering the presence of PCN species constitute a threat for potato production, intensive sampling and monitoring of this plant pathogen should be conducted to reduce and prevent the spread into new areas. The development of management practices that involves the evaluation of resistant varieties for populations that tested positive for PCN, as well as other practices such as crop rotations, trap crops, biofumigants, biocontrol agents among others that have shown to be effective for other *G*. *pallida* populations worldwide, are also a crucial step to reduce population densities of PCN in Colombia.
